# Validation of clinical‐grade whole genome sequencing reproduces cytogenetic analysis and identifies mutational landscape in newly‐diagnosed multiple myeloma patients: A pilot study from the 100,000 Genomes Project

**DOI:** 10.1002/jha2.276

**Published:** 2021-08-26

**Authors:** Oliver C. Lomas, Sarah Gooding, Maite Cabes, Helene Dreau, Edward Wilson, Paolo Polzella, Genomics England Research Consortium, Karthik Ramasamy, Angela D. Hamblin

**Affiliations:** ^1^ Department of ClinicalHaematology John Radcliffe and Churchill Hospitals Oxford University Hospitals NHS Trust Oxford UK; ^2^ Genomics England London UK

## Abstract

Multiple myeloma is characterized by chromosomal abnormalities and genetic variation, which may inform prognosis and guide treatment. This pilot study sought to examine the feasibility of incorporating Whole Genome Sequencing (WGS) alongside the routine laboratory evaluation of 14 patients with newly diagnosed multiple myeloma who had enrolled in the 100,000 Genomes Project. In all 14 cases, WGS data could be obtained in a timely fashion within existing clinical frameworks in a tertiary hospital setting. The data not only replicated standard‐of‐care FISH analysis of chromosomal abnormalities but also provided further chromosomal and molecular genetic insights that may influence patient management.

## 1 INTRODUCTION

Multiple myeloma (MM) is caused by the malignant proliferation of plasma cells. Complex karyotypes that comprise both numerical and structural chromosomal abnormalities are found almost universally in MM. Hyperdiploid cases are driven by numerical gains of chromosomes, whereas in non‐hyperdiploid cases, the majority of cells carry oncogenic translocations with some chromosomal losses [1]. Detection of these abnormalities by Fluorescent in situ Hybridization (FISH) informs prognostication in myeloma [2] and perhaps therapeutic choice [3]. Furthermore, there are frequent mutations in individual genes that have been identified through whole genome sequencing (WGS) of malignant plasma cells [4]. The presence of the mutated genes may be used for prognostication, the detection of measurable residual disease (MRD), and even to indicate specific therapies [5]. The 100,000 Genome Project led by Genomics England aimed to develop a platform that permits the integration of massively parallel WGS data into clinical practice [6]. The aim of our study was to describe the feasibility and utility of delivering such detailed data into current practice and begin to validate the structural genomic abnormalities identified by standard‐of‐care FISH analysis.

## 2 METHODS

Fourteen patients with newly diagnosed multiple myeloma consented for WGS at Oxford University Hospitals NHS Trust between June 2017 and April 2018. Plasma cells were selected by MACS^®^ magnetic bead CD138^+^ cell enrichment from surplus bone marrow aspirate at diagnosis. Genomic DNA was extracted locally to a minimum yield of 0.5 μg. Germline samples were derived from peripheral blood that was taken simultaneously with bone marrow aspiration. Plasma cell leukemia was excluded by examination of a blood film from the peripheral blood sample. Baseline patient characteristics are shown in Table 1.

**TABLE 1:** Baseline data of patient and sample characteristics

**Median Age at Diagnosis**	76 years	
**Sex**	7 male 7 female	
**Immunoglobulin heavy chain class**	IgG = 8 IgA = 2 Light chain only = 4	
**Immunoglobulin light chain class**	κ = 9 λ = 5	
**International Staging System (ISS) score (Frequency)**	Stage 1	3
	Stage 2	3
	Stage 3	9
**Plasma cell morphological purity**	78 – 99%	
**WGS Depth of Sequencing**	Germline – 32.99x	
	Tumour – 99.87x	

At least 100 cells were scored for each FISH probe to detect the following variants, according to criteria from the International Myeloma Working Group (IMWG [2]): *IGH‐FGFR3* t(4;14)(p16;q32), *IGH‐MAF* t(14;16)(q32;q23), *IGH‐CCND1* t(11;14)(q13;q32), *IGH‐MAFB* t(14;20)(q3;q12), 17p13 deletions, Chromosome 1p deletion, or 1q gain. WGS of tumor and germline DNA samples was performed on a HiSeqX sequencer (Illumina, Inc., San Diego, CA, USA) according to standard operating procedures and analyzed using the bioinformatics pipeline developed for the Genomics England Main Programme Analysis version 1.9. Sequencing reads were aligned to the Human Genome Assembly GRCh38 with ISAAC (version iSAAC‐03.16.02.19); small variant calling together with tumor‐normal subtraction was performed using Strelka (version 2.4.7), and Manta 0.28.0 and Canvas 1.3.1 for detection of somatic CNAs (Illumina, Inc., San Diego, CA, USA). Data returned from Genomics England in the Whole Genome Analysis comprised all somatic variants (produced following germline variant subtraction), whose consequence was predicted to alter the protein‐coding region or adjacent splice sites as well as known or likely pathogenic germline variants in genes associated with cancer susceptibility.

## 3 RESULTS AND DISCUSSION

In total, seven FISH probes were applied successfully to the plasma cells of 14 patients. Out of 98 individual tests, 13 significant cytogenetic abnormalities were detected. Concordance between FISH and WGS was 100%, with no false negative or false positive conflicts.

Table 2 provides a summary of the observations in our cohort of newly diagnosed patients with multiple myeloma where WGS provided supplementary information to standard‐of‐care FISH analysis of plasma cells. WGS was able to provide significant insight into structural genomic abnormalities that were not fully appreciated by a pre‐defined panel of FISH probes. One case with no apparent cytogenetic abnormality was revealed to harbor a deletion of Chromosome 1p31.1;p12 by WGS. The deleted region did not include the locus that covers *CDKN2C* (1p32.3), which was where the FISH probe was centered, but the deleted region does include *FAM46C* (1p12). Deletion or mutation of *FAM46C* has been associated with a worse prognosis [7]. This chromosomal abnormality constituted the sole marker of poor prognosis in this individual case. There is nothing to suggest that an appropriate FISH probe would have missed this deletion, but the example highlights the benefit of an unbiased WGS assessment of chromosomal abnormalities against a limited FISH panel. Similarly, the FISH analysis lacked a marker for Chromosome 13 deletions, three of which were identified by WGS.

**TABLE 2:** Summary of structural chromosomal data provided by WGS supplementary to the panel of standard‐of‐care FISH probes

*Fluorescence in situ Hybridisation*	*Whole Genome Sequencing*
Probe for Del 1p32 (*CDKN2c*) – nil observed	In one case, del(1p31.1;p12) detected, which encompasses a region involving *FAM46c* at 1p12. Deletion or mutation is a marker of poor risk [7]
No probe for deletion of Chromosome 13	Identified three cases of deletion of Chromosome 13
*IGH* partner translocation suspected not identified	Partner identification resolved in each case: t(8;14)(q24.21;q32.33) – *MYC/IGH* juxtaposition reported poor prognosis [12] t(14;20)(q32.33;q13.12) – Additional amplification of 20q13.12 carries loci of at least two potential oncogenes (*WISP2*, *UBE2C*) [8]. t(2;14) and Indel of 14q32.33 may account for inconclusive FISH result

Table 3 describes the comparison of FISH and WGS in relation to *IGH* translocations. FISH identified five known translocations involving the *IGH* locus at 14q32, with three further instances with unidentifiable translocation partners. WGS agreed with the five instances specified by FISH and identified the three other translocations. No *IGH* translocations were suggested by WGS that were not identified or inferred by FISH analysis. Of the two suspected *IGH* partners confirmed by WGS, one was for t(8;14), which juxtaposes *MYC* with *IGH*, a known poor prognostic marker; and the other was a novel t(4;20) breakpoint (20q13), which carries loci of at least two genes with oncogenic potential (*WISP2*, *UBE2C*) [8]. The third case of a translocation involving 14q32 with an unknown partner by FISH correlated with an Indel region of 14q32.33 and t(2;14 translocation) by WGS, which may account for the inconclusive result in this case. These observations highlight the utility of the unbiased analysis provided by WGS.

**TABLE 3:** Translocations involving 14q32.33 locus of *IGH* identified by WGS in addition to defined FISH probes according to IMWG criteria

*Case*	*Translocation*	*Comparative analysis by FISH and WGS*
1	t(14;19)(q32.33;p13.3)	Similar translocations implicated in B‐cell malignancy [13]
3	t(1;14)(p35.3;q32.33)	Not previously described but t(1;14)(p35.**2**;32.33) described in a myeloma cell line [14]
4	t(1;14)(q21.3;q32.33)	Not described but 1q21.3 locus does cover soluble IL6‐Receptor (OMIM : 614689)
7	t(8;14)(q24.21;q32.33)	* Translocation juxtaposes *MYC* and *IGH*. Poor prognostic marker. [12]
8	t(2;14) and InDel 14q32.33	* Translocation partner not identified by FISH, but t(2;14) and insertion Deletion (InDel) at 14q32.33 on WGS
12	t(14;20)(q32.33;q13.12)	* Translocation t(14;20)(q32;q12) IGH/MAFB associated with poor prognosis. Amplification of 20q13.12 carrying loci of at least two genes with oncogenic potential (*WISP2*, *UBE2C*) [8].
14	t(10;14)(q26.11;q32.33)	Not previously described. 10q26.11 encompasses *FGFR2*, mutations of which have been described in MM [15].

* denotes translocation suggested by FISH probes but not identified by that assay – three out of 4 seven cases. Patients 2,5,6,8‐11,13 had no evidence of a translocation involving 14q32.33.

WGS removes the bias of a predetermined panel of probes, as used in FISH. The data provided were analyzed using a generic cancer pipeline that comprised a list of 200 genes known to be relevant in all cancers. A virtual panel of 22 genes relevant to risk stratification and potential precision drug therapy was applied to the 14 cases (Table S1). One case involved a frameshift mutation of the tumor suppressor gene *RB1*, which is found at 13q14.2. This loss of function mutation, at a Variant Allele Frequency (VAF) of 0.47, was found in a case where WGS had also identified a loss of one allele of Chromosome 13, which may represent a sub‐clone that comprises prognostically significant bi‐allelic inactivation of *RB1*. It was not possible to confirm this with the pipeline used in this case series but may be achieved with a refined bioinformatic approach. The virtual panel identified mutations in eight of the 14 cases that were potentially amenable to targeted drug therapy. For example, the MyDrug study (ClinicalTrials.gov : NCT03732703) has been devised to use selective therapies for patients with relapsed‐refractory MM with mutations in *NRAS*, *KRAS*, *BRAF* V600E, *CDK*, *FGFR3*, and *IDH2*. However, relevant trial information is required to determine the benefit of such precision therapy in the setting of newly diagnosed myeloma. A detailed description of all the observations based on the virtual gene panel in each of the 14 cases is given in Table S2. Individual gene mutation data may be used to identify patient‐specific *IGH* V‐D‐J rearrangements as markers of Measurable Residual Disease (MRD) throughout the clinical course of myeloma management [9]. Compared to sequencing technologies, FISH may fail to identify prognostically significant structural variants in the *MYC* oncogene [10].

This pilot study demonstrates the ability to incorporate WGS into routine clinical practice using pre‐existing laboratory infrastructure with a centralized resource for massively parallel sequencing. Numerous advantages may be envisaged with a sequencing approach to the genomic characterization of MM. All FISH probe results (positive and negative) were replicated by WGS, using sub‐optimal remnant samples, within existing clinical structures at a turnaround time of as quick as 14 days. This turnaround time from sample preparation to sequencing and a standardized report could be achieved in a time frame comparable to standard‐of‐care FISH. WGS was able to provide further structural genomic information that was beyond the scope of the FISH panel. Additional FISH probes could be employed to detect the structural genomic information provided by WGS. However, this would increase the cost and time required for the analysis, compared to the fixed cost per sample of WGS. The use of the widely performed technique of DNA extraction may allow improved accessibility to genetic risk stratification when performed by WGS, compared to centralized myeloma FISH expertise, which may add further delay in assessment.

A limitation of bulk, massively parallel WGD is the potential for missing small, sub‐clonal structural or single‐nucleotide variants. FISH analysis is limited to a comparatively small number of cells but can identify subclones with structural variants that may be missed by WGS. This may be mitigated by using improved bioinformatic pipelines or by single‐cell sequencing technologies that are able to detect accurate clonal fractions of SNVs and copy number aberrations and defining their sub‐clonal structure. The cost of massively parallel sequencing is falling precipitously but is still a significant consideration, especially as the subsequent assessment of somatic genomes at disease progression may guide therapeutic choice [11].

## 4 CONCLUSION

This study demonstrates the practical feasibility of incorporating WGS into existing diagnostic laboratory frameworks. Furthermore, this pilot study provides some insight into the validity and utility of WGS compared to standard‐of‐care FISH analysis in myeloma. For precision medicine to become available to patients, specific agents are likely required to target the disease at many levels of intratumoral heterogeneity, including gene expression and epigenetics. Massively parallel sequencing not only yields information about individual genes and chromosomal structure, but also represents a platform whereby such additional sequencing information may be incorporated to define more closely this highly heterogenous malignancy and thus target it more effectively.

*Genomics England Research Consortium (GERC)*

Ambrose J. C. ^1^, Arumugam P.^1^ iD, Baple E. L. ^1^ iD, Bleda M.^1^iD, Boardman‐Pretty F.^1,2^ iD, Boissiere J. M.^1^, Boustred C. R.^1^, Brittain H.^1^ iD, Caulfield M. J.^1,2^, Chan G. C.^1^, Craig C. E. H.^1^iD, Daugherty L. C.^1^iD, de Burca A.^1^, Devereau, A.^1^, Elgar G.^1,2^ iD, Foulger R. E.^1^iD, Fowler T.^1^iD, Furió‐Tarí P.^1^iD, Hackett J. M.^1^, Halai D.^1^, Hamblin A.^1^, Henderson S.^1,2^, Holman J. E.^1^, Hubbard T. J. P.^1^iD, Ibáñez K.^1,2^ iD, Jackson R. ^1^ iD, Jones L. J. ^1,2^, Kasperaviciute D.^1,2^, Kayikci M.^1^iD, Lahnstein L.^1^, Lawson K.^1^iD, Leigh S. E. A.^1^iD, Leong I. U. S.^1^iD, Lopez F. J.^1^, Maleady‐Crowe F.^1^, Mason J.^1^iD, McDonagh E. M.^1,2^ iD, Moutsianas L.^1,2^ iD, Mueller M.^1,2^ iD, Murugaesu N. ^1^, Need A. C.^1,2^ iD, Odhams C. A.^1^iD, Patch C.^1,2^ iD, Perez‐Gil D.^1^, Pereira M. B.^1^ iD, Polychronopoulos D.^1^iD, Pullinger J.^1^iD, Rahim T.^1^iD, Rendon A.^1^iD, Riesgo‐Ferreiro P.^1^ iD, Rogers T.^1^, Ryten M.^1^, Savage K.^1^, Sawant K. ^1^, Scott R. H.^1^, Siddiq A.^1^iD, Sieghart A.^1^iD, Smedley D.^1,2^, Smith K. R.^1,2^ iD, Sosinsky A.^1,2^ iD, Spooner W.^1^iD, Stevens H. E.^1^iD, Stuckey A.^1^iD, Sultana R. ^1^, Thomas E. R. A.^1,2^ iD, Thompson S. R.^1^iD, TregiDgo C.^1^, Tucci A.^1,2^ iD, Walsh E.^1^iD, Watters, S. A.^1^iD, Welland M. J.^1^, Williams E.^1^iD, Witkowska K.^1,2^, Wood S. M.^1,2^, Zarowiecki M.^1^ iD.

1. Genomics England, London, UK

2. William Harvey Research Institute, Queen Mary University of London, London, UK

## AUTHOR CONTRIBUTIONS

OCL, SG, and AH designed the research, analyzed the data and OCL wrote the paper; OCL, SG, MC, HD, EW, PP, KR and AH performed the research and/or contributed patient samples and associated data. All authors read and agreed to the final version of the manuscript. GERC provided the framework for sequencing and analysis. GERC have been consulted about the manuscript and agree to its submission.

## CONFLICT OF INTEREST

The authors declare no conflict of interest.

## Supporting information

Supporting information

## References

[1] Wuilleme S , Robillard N , Lode L , Magrangeas F , Beris H , Harousseau JL , et al. Ploidy, as detected by fluorescence in situ hybridization, defines different subgroups in multiple myeloma. Leukemia. 2005;19:275–78. 15538401 10.1038/sj.leu.2403586

[2] Chng WJ , Dispenzieri A , Chim CS , Fonseca R , Goldschmidt H , Lentzsch S , et al. IMWG consensus on risk stratification in multiple myeloma. Leukemia. 2014;28:269–77. 23974982 10.1038/leu.2013.247

[3] Kumar SK , Harrison SJ , Cavo M , de la Rubia J , Popat R , Gasparetto C , et al. Venetoclax or placebo in combination with bortezomib and dexamethasone in patients with relapsed or refractory multiple myeloma (BELLINI): a randomised, double‐blind, multicentre, phase 3 trial. Lancet Oncol. 2020;21:1630–42. 33129376 10.1016/S1470-2045(20)30525-8

[4] Chapman MA , Lawrence MS , Keats JJ , Cibulskis K , Sougnez C , Schinzel AC , et al. Initial genome sequencing and analysis of multiple myeloma. Nature. 2011;471:467–72. 21430775 10.1038/nature09837 PMC3560292

[5] Gonzalez‐Calle V , Keane N , Braggio E , Fonseca R . Precision Medicine in Myeloma: Challenges in Defining an Actionable Approach. Clin Lymphoma Myeloma Leuk 2017;17:621–30. 28743429 10.1016/j.clml.2017.06.021

[6] Caulfield M , Davies J , Dennys M , Elbahy L , Fowler T , Hill S , et al. The 100,000 Genomes Project Protocol . 2017.

[7] Boyd KD , Ross FM , Walker BA , Wardell CP , Tapper WJ , Chiecchio L , et al. Mapping of chromosome 1p deletions in myeloma identifies *FAM46C* at 1p12 and *CDKN2C* at 1p32.3 as being genes in regions associated with adverse survival. Clin Cancer Res. 2011;17:7776–84. 21994415 10.1158/1078-0432.CCR-11-1791 PMC5751883

[8] Carrasco DR , Tonon G , Huang Y , Zhang Y , Sinha R , Feng B , et al. High‐resolution genomic profiles define distinct clinico‐pathogenetic subgroups of multiple myeloma patients. Cancer Cell. 2006;9:313–25. 16616336 10.1016/j.ccr.2006.03.019

[9] Anderson KC , Auclair D , Kelloff GJ , Sigman CC , Avet‐Loiseau H , Farrell AT et al. The Role of Minimal Residual Disease Testing in Myeloma Treatment Selection and Drug Development: Current Value and Future Applications. Clin Cancer Res. 2017;23:3980–93. 28428191 10.1158/1078-0432.CCR-16-2895

[10] Sharma N , Smadbeck JB , Abdallah N , Zepeda‐Mendoza C , Binder M , Pearce KE , et al. The prognostic role of MYC structural variants identified by NGS and FISH in multiple myeloma. Clin Cancer Res. 2021. 10.1158/1078-0432.CCR-21-0005 PMC8738776 34233962

[11] Gooding S , Ansari‐Pour N , Towfic F , Ortiz Estevez M , Chamberlain PP , Tsai KT , et al. Multiple cereblon genetic changes are associated with acquired resistance to lenalidomide or pomalidomide in multiple myeloma. Blood. 2021;137:232–37. 33443552 10.1182/blood.2020007081 PMC7893409

[12] Walker BA , Wardell CP , Brioli A , Boyle E , Kaiser MF , Begum DB , et al. Translocations at 8q24 juxtapose *MYC* with genes that harbor superenhancers resulting in overexpression and poor prognosis in myeloma patients. Blood Cancer J. 2014;4:e191. 24632883 10.1038/bcj.2014.13 PMC3972699

[13] Russell LJ , De Castro DG , Griffiths M , Telford N , Bernard O , Panzer‐Grumayer R , et al. A novel translocation, t(14;19)(q32;p13), involving IGH@ and the cytokine receptor for erythropoietin. Leukemia. 2009;23:614–17. 18818706 10.1038/leu.2008.250

[14] Hayami Y , Iida S , Nakazawa N , Hanamura I , Kato M , Komatsu H , et al. Inactivation of the *E3/LAPTm5* gene by chromosomal rearrangement and DNA methylation in human multiple myeloma. Leukemia. 2003;17:1650–57. 12886255 10.1038/sj.leu.2403026

[15] Lohr JG , Stojanov P , Carter SL , Cruz‐Gordillo P , Lawrence MS , Auclair D , et al. Widespread genetic heterogeneity in multiple myeloma: implications for targeted therapy. Cancer Cell. 2014;25:91–101. 24434212 10.1016/j.ccr.2013.12.015 PMC4241387

