# Supplementary material for: Validation of clinical‐grade whole genome sequencing reproduces cytogenetic analysis and identifies mutational landscape in newly‐diagnosed multiple myeloma patients: A pilot study from the 100,000 Genomes Project
Source: EJHaem. 2021 Aug 26;2(4):809–12. doi: 10.1002/jha2.276 (PMC9175844; doi:10.1002/jha2.276)
Supplement: Supplementary file 1 — Supporting information [file JHA2-2-809-s001.docx]

**Supplementary Table 1 (S1) - Virtual panel of genes relevant to multiple myeloma**

|  | **Cytogenetic Location** | **Gene function** | **Relevance to multiple myeloma** |
| --- | --- | --- | --- |
| *ATM* | 11q22.3 | Encodes cell cycle checkpoint kinase involved in the repair of DNA damage by homologous recombination | Found in a minority of cases of MM but in up to 40% of cases of mantle cell lymphoma. (Lohr*, et al* 2014) . May be amenable to PARP inhibition therapy. |
| *BRAF* | 7q34 | Encodes for a serine/threonine kinase that activates the MEK/ERK signalling pathway. | Established driver mutation in myeloma associated with t(14:16) (Bolli*, et al* 2018, Walker*, et al* 2018). Nature of *BRAF* mutation (e.g. V600E or not) influences whether therapeutic intervention. |
| *CCND1* | 11q13.3 | Encodes Cyclin D1, which couples extracellular growth signals to cell cycle entry. | *CCND1* is a target of, and only significantly mutated in, the t(11;14) translocation. (Walker*, et al* 2018)Walker et al 2018 (Blood) |
| *CDKN1B* | 12p13.1 | A tumour suppressor gene that encodes a cyclin-dependent kinase inhibitor. | Infrequently mutated in myeloma. Associated with hyperdiploid cytogenetics. Infrequently lost through copy number loss or mutations. (Walker*, et al* 2018) |
| *CDKN2C* | 1p32.3 | A tumour suppressor gene that encodes a cyclin-dependent kinase inhibitor. | Lost in del 1q cytogenetic abnormalities, through rarely by mutation. Lohr et al 2014 (Cancer Cell); Walker et al 2018 (Blood) |
| *CYLD* | 16q12.1 | A tumour suppressor gene encoding a deubiquitinase enzyme | Mutations of *CYLD* confer a poor prognosis in myeloma (Jenner*, et al* 2007) |
| *FAM46C* | 1p12 | Tumour suppressor gene that encodes a nucleotidyl-transferase that enhances mRNA stability and gene expression. | Inactivating mutations of *FAM46C* are drivers of myeloma (Mroczek*, et al* 2017) |
| *FGFR3* | 4p16.3 | Encodes a member of the fibroblast growth factor receptor family. | A target of t(4;14) translocation, mutated in about 1/3 of t(4;14) (Walker*, et al* 2018) |
| *IDH2* | 15q26.1 | Encodes a mitochondrial enzyme that catalyses the oxidative decarboxylation of isocitrate to α-ketoglutarate. Mutant IDH enzymes have gain-of-function mutations activity. | *IDH2* represents a CHIP mutation but is described rarely in myeloma (Lohr*, et al* 2014). FDA-approved *IDH2* inhibitors are available, which may trial option. |
| *IGF1R* | 15q26.3 | A tyrosine kinase that opposes apoptosis. | *IGF1R* encodes a tyrosine kinase. Missense mutations of *IGF1R* account in 1-2% of cases of myeloma (Lohr*, et al* 2014). |
| *IRF4* | 6p25.3 | Encodes a transcription factor that regulates interferon-inducible genes. *IRF4* is a target of MYC transactivation. | Associated with t(11;14) cytogenetics. Potential therapeutic target (Agnarelli*, et al* 2018). |
| *KRAS* | 12p12.1 | Encodes a GTPase that regulates signalling via  MAPK and PI3K. | Mutated in 25-30% of cases of myeloma as well as many other malignancies (Chapman*, et al* 2011, Lohr*, et al* 2014, Walker*, et al* 2018). |
| *MAF* | 16p23.2 | Transcription factor | *MAF* is a target of, and only significantly mutated in, the t(14;16) translocation. Only significantly mutated in t(14;16), increased activity associated with bortezomib resistance (Qiang*, et al* 2016, Walker*, et al* 2018) |
| *MAFB* | 20q12 | Transcription factor | *MAFB* is a target of, and only significantly mutated in, the t(14;20) translocation. Only significantly mutated in t(14;20), increased activity associated with bortezomib resistance (Walker*, et al* 2018) |
| *MAX* | 14q23.3 | Transcription factor | Loss of function mutations are found in about 1% of cases. Deletion of 14q with a mutation in MAX results in bi-allelic inactivation of the gene (analogous to del 17p and *TP53*). (Lohr*, et al* 2014) |
| *NRAS* | 1p13.2 | GTPase that regulates MAPK and PI3K signalling pathways. | Mutated in 25-30% of cases of myeloma as well as many other malignancies (Chapman*, et al* 2011, Lohr*, et al* 2014, Walker*, et al* 2018). |
| *PPM1D* | 17q23.3 | A serine-threonine phosphatase which negatively regulates the cellular stress and DNA damage response. | *PPM1D* mutations are enriched in patients with therapy-related myeloid neoplasms and may be of relevance following stem cell transplantation. |
| *PTPN11* | 12q24.13 | *PTPN11* encodes a protein phosphatase with the E76K mutation known to be oncogenic | Associated with hyperdiploid cytogenetics.(Lohr*, et al* 2014) |
| *RB1* | 13q14.2 | The first identified) tumour suppressor gene | Rarely mutated in myeloma but is lost in Del(13q) (Chavan*, et al* 2017, Lohr*, et al* 2014, Walker*, et al* 2018) |
| *TGDS* | 13q32.1 | Metabolic enzyme | Mutations associated with worse prognosis by univariate analysis (Bolli*, et al* 2014). |
| *TP53* | 17p13.1 | Encodes a tumour suppressor protein that regulates gene expression in response to cellular stress and DNA damage | Loss-of-function mutations lead to genome instability and enhanced cell survival. Often associated with higher risk disease, chemotherapy resistance, and poor prognosis.  (Chapman*, et al* 2011, Walker*, et al* 2019) |
| *TRAF3* | 14q32.32 | TNF receptor associated factor that participates in CD40 signal transduction in B lymphocytes. | Rarely mutated in myeloma but is lost in del(14q). Associated with worse prognosis by univariate analysis. Walker (Mambetsariev*, et al* 2016, Walker*, et al* 2018) |

**Supplementary Table 2 (S2)**

Summary of significant gene mutations by case.

| ***Patient*** | ***Mutation*** | ***VAF*** | ***Notes*** |
| --- | --- | --- | --- |
| 1 | *KRAS* G34C | 0.41 | Missense mutation |
| 2 | *FGFR3* Y241S | 0.28 | Missense mutation |
| 3 | *CYLD* | 0.2 0.4 | Three mutations : 2 missense, 1 frameshift |
| 4 | *KRAS* G13D | 0.05 | Missense mutation |
|  | *FAM46C* | 0.17 | Frameshift variants |
|  | *PTPN11* G503V | 0.09 | Missense gain of function mutation - Likely oncogenic. Described previously in MM (Lohr*, et al* 2014) |
| 5 | Nil on Virtual Panel |  |  |
| 6 | Nil on Virtual Panel |  |  |
| 7 | *NRAS* G61L  *FAM46C* I276del | 0.42  0.98 | Missense mutation  Nil on FISH |
| 8 | *NRAS* Q61R  *RB1* 1769delG | 0.26  0.47 | *RB1* associated with a deletion of Chromosome may represent bi-allelic inactivation (Chavan*, et al* 2017) |
| 9 | *NRAS* Q61H | 0.41 | Missense mutation  Nil on FISH |
| 10 | *NRAS* Q61K | 0.4 | Missense mutation  Nil on FISH |
| 11 | *NRAS* G13D  *FAM46C* N84K | 0.46  0.17 | Missense mutations  Nil on FISH |
| 12 | *BRAF* V471F  *BRAF* G466E | 0.04  0.06 | Neither *BRAF* mutation V600E |
| 13 | *KRAS* Q61H | 0.2 | Missense mutations  Nil on FISH |
| 14 | *CYLD* | 0.09, 0.24, 0.38 | Two missense mutations and one frameshift mutation |

**Supplementary References**

Agnarelli, A., Chevassut, T. & Mancini, E.J. (2018) IRF4 in multiple myeloma-Biology, disease and therapeutic target. *Leuk Res,* **72,** 52-58.

Bolli, N., Avet-Loiseau, H., Wedge, D.C., Van Loo, P., Alexandrov, L.B., Martincorena, I., Dawson, K.J., Iorio, F., Nik-Zainal, S., Bignell, G.R., Hinton, J.W., Li, Y., Tubio, J.M., McLaren, S., S, O.M., Butler, A.P., Teague, J.W., Mudie, L., Anderson, E., Rashid, N., Tai, Y.T., Shammas, M.A., Sperling, A.S., Fulciniti, M., Richardson, P.G., Parmigiani, G., Magrangeas, F., Minvielle, S., Moreau, P., Attal, M., Facon, T., Futreal, P.A., Anderson, K.C., Campbell, P.J. & Munshi, N.C. (2014) Heterogeneity of genomic evolution and mutational profiles in multiple myeloma. *Nature Communications,* **5,** 2997.

Bolli, N., Biancon, G., Moarii, M., Gimondi, S., Li, Y., de Philippis, C., Maura, F., Sathiaseelan, V., Tai, Y.T., Mudie, L., O'Meara, S., Raine, K., Teague, J.W., Butler, A.P., Carniti, C., Gerstung, M., Bagratuni, T., Kastritis, E., Dimopoulos, M., Corradini, P., Anderson, K.C., Moreau, P., Minvielle, S., Campbell, P.J., Papaemmanuil, E., Avet-Loiseau, H. & Munshi, N.C. (2018) Analysis of the genomic landscape of multiple myeloma highlights novel prognostic markers and disease subgroups. *Leukemia,* **32,** 2604-2616.

Chapman, M.A., Lawrence, M.S., Keats, J.J., Cibulskis, K., Sougnez, C., Schinzel, A.C., Harview, C.L., Brunet, J.P., Ahmann, G.J., Adli, M., Anderson, K.C., Ardlie, K.G., Auclair, D., Baker, A., Bergsagel, P.L., Bernstein, B.E., Drier, Y., Fonseca, R., Gabriel, S.B., Hofmeister, C.C., Jagannath, S., Jakubowiak, A.J., Krishnan, A., Levy, J., Liefeld, T., Lonial, S., Mahan, S., Mfuko, B., Monti, S., Perkins, L.M., Onofrio, R., Pugh, T.J., Rajkumar, S.V., Ramos, A.H., Siegel, D.S., Sivachenko, A., Stewart, A.K., Trudel, S., Vij, R., Voet, D., Winckler, W., Zimmerman, T., Carpten, J., Trent, J., Hahn, W.C., Garraway, L.A., Meyerson, M., Lander, E.S., Getz, G. & Golub, T.R. (2011) Initial genome sequencing and analysis of multiple myeloma. *Nature,* **471,** 467-472.

Chavan, S.S., He, J., Tytarenko, R., Deshpande, S., Patel, P., Bailey, M., Stein, C.K., Stephens, O., Weinhold, N., Petty, N., Steward, D., Rasche, L., Bauer, M., Ashby, C., Peterson, E., Ali, S., Ross, J., Miller, V.A., Stephens, P., Thanendrarajan, S., Schinke, C., Zangari, M., van Rhee, F., Barlogie, B., Mughal, T.I., Davies, F.E., Morgan, G.J. & Walker, B.A. (2017) Bi-allelic inactivation is more prevalent at relapse in multiple myeloma, identifying *RB1* as an independent prognostic marker. *Blood Cancer Journal,* **7,** e535.

Jenner, M.W., Leone, P.E., Walker, B.A., Ross, F.M., Johnson, D.C., Gonzalez, D., Chiecchio, L., Dachs Cabanas, E., Dagrada, G.P., Nightingale, M., Protheroe, R.K., Stockley, D., Else, M., Dickens, N.J., Cross, N.C., Davies, F.E. & Morgan, G.J. (2007) Gene mapping and expression analysis of 16q loss of heterozygosity identifies WWOX and CYLD as being important in determining clinical outcome in multiple myeloma. *Blood,* **110,** 3291-3300.

Lohr, J.G., Stojanov, P., Carter, S.L., Cruz-Gordillo, P., Lawrence, M.S., Auclair, D., Sougnez, C., Knoechel, B., Gould, J., Saksena, G., Cibulskis, K., McKenna, A., Chapman, M.A., Straussman, R., Levy, J., Perkins, L.M., Keats, J.J., Schumacher, S.E., Rosenberg, M., Multiple Myeloma Research, C., Getz, G. & Golub, T.R. (2014) Widespread genetic heterogeneity in multiple myeloma: implications for targeted therapy. *Cancer Cell,* **25,** 91-101.

Mambetsariev, N., Lin, W.W., Stunz, L.L., Hanson, B.M., Hildebrand, J.M. & Bishop, G.A. (2016) Nuclear TRAF3 is a negative regulator of CREB in B cells. *Proc Natl Acad Sci U S A,* **113,** 1032-1037.

Mroczek, S., Chlebowska, J., Kulinski, T.M., Gewartowska, O., Gruchota, J., Cysewski, D., Liudkovska, V., Borsuk, E., Nowis, D. & Dziembowski, A. (2017) The non-canonical poly(A) polymerase FAM46C acts as an onco-suppressor in multiple myeloma. *Nature Communications,* **8,** 619.

Qiang, Y.W., Ye, S., Chen, Y., Buros, A.F., Edmonson, R., van Rhee, F., Barlogie, B., Epstein, J., Morgan, G.J. & Davies, F.E. (2016) MAF protein mediates innate resistance to proteasome inhibition therapy in multiple myeloma. *Blood,* **128,** 2919-2930.

Walker, B.A., Mavrommatis, K., Wardell, C.P., Ashby, T.C., Bauer, M., Davies, F., Rosenthal, A., Wang, H., Qu, P., Hoering, A., Samur, M., Towfic, F., Ortiz, M., Flynt, E., Yu, Z., Yang, Z., Rozelle, D., Obenauer, J., Trotter, M., Auclair, D., Keats, J., Bolli, N., Fulciniti, M., Szalat, R., Moreau, P., Durie, B., Stewart, A.K., Goldschmidt, H., Raab, M.S., Einsele, H., Sonneveld, P., San Miguel, J., Lonial, S., Jackson, G.H., Anderson, K.C., Avet-Loiseau, H., Munshi, N., Thakurta, A. & Morgan, G. (2019) A high-risk, Double-Hit, group of newly diagnosed myeloma identified by genomic analysis. *Leukemia,* **33,** 159-170.

Walker, B.A., Mavrommatis, K., Wardell, C.P., Ashby, T.C., Bauer, M., Davies, F.E., Rosenthal, A., Wang, H., Qu, P., Hoering, A., Samur, M., Towfic, F., Ortiz, M., Flynt, E., Yu, Z., Yang, Z., Rozelle, D., Obenauer, J., Trotter, M., Auclair, D., Keats, J., Bolli, N., Fulciniti, M., Szalat, R., Moreau, P., Durie, B., Stewart, A.K., Goldschmidt, H., Raab, M.S., Einsele, H., Sonneveld, P., San Miguel, J., Lonial, S., Jackson, G.H., Anderson, K.C., Avet-Loiseau, H., Munshi, N., Thakurta, A. & Morgan, G.J. (2018) Identification of novel mutational drivers reveals oncogene dependencies in multiple myeloma. *Blood,* **132,** 587-597.
